# The rationale and design of Insight into Nephrotic Syndrome: Investigating Genes, Health and Therapeutics (INSIGHT): a prospective cohort study of childhood nephrotic syndrome

**DOI:** 10.1186/1471-2369-14-25

**Published:** 2013-01-26

**Authors:** Neesha Hussain, J Anastasia Zello, Jovanka Vasilevska-Ristovska, Tonny M Banh, Viral P Patel, Pranali Patel, Christopher D Battiston, Diane Hebert, Christoph P B Licht, Tino D Piscione, Rulan S Parekh

**Affiliations:** 1 Division of Nephrology, The Hospital for Sick Children, 555 University Avenue, M5G 1X8, Toronto, ON, Canada; 2 Child Health Evaluative Sciences, Research Institute, The Hospital for Sick Children, 555 University Avenue, M5G 1X8, Toronto, ON, Canada; 3 University Health Network, 190 Elizabeth Street, M5G 2C4, Toronto, ON, Canada; 4 University of Toronto, 27 King’s College Circle, M5S 1A1, Toronto, ON, Canada; 5 Dalla Lana School of Public Health, University of Toronto, 27 King’s College Circle, M5S 1A1, Toronto, ON, Canada; 6 University of Saskatchewan, 105 Administration Place, S7N 5A2, Saskatoon, SK, Canada; 7 Genetic and Genome Biology, Research Institute, The Hospital for Sick Children, 555 University Avenue, M5G 1X8, Toronto, ON, Canada

**Keywords:** Children, Nephrotic syndrome, Cohort, Chronic kidney disease, FSGS, Minimal change disease, Study protocol

## Abstract

**Background:**

Nephrotic syndrome is one of the most commonly diagnosed kidney diseases in childhood and its progressive forms can lead to chronic kidney disease (CKD) and/or end-stage renal disease (ESRD). There have been few longitudinal studies among a multi-ethnic cohort to determine potential risk factors influencing disease susceptibility, treatment response, and progression of nephrotic syndrome. Temporal relationships cannot be studied through cross-sectional study design. Understanding the interaction between various factors is critical to developing new strategies for treating children with kidney disease. We present the rationale and the study design of a longitudinal cohort study of children with nephrotic syndrome, the Insight into Nephrotic Syndrome: Investigating Genes, Health and Therapeutics (INSIGHT) study. The specific aims are to determine: 1) socio-demographic, environmental, and genetic factors that influence disease susceptibility; 2) rates of steroid treatment resistance and steroid treatment dependence, and identify factors that may modify treatment response; 3) clinical and genetic factors that influence disease susceptibility and progression to CKD and ESRD; and 4) the interaction between the course of illness and socio-demographic, environmental, and clinical risk factors.

**Methods/design:**

INSIGHT is a disease-based observational longitudinal cohort study of children with nephrotic syndrome. At baseline, participants complete questionnaires and provide biological specimen samples (blood, urine, and toenail clippings). Follow-up questionnaires and repeat biological specimen collections are performed annually for up to five years.

**Discussion:**

The proposed cohort will provide the structure to test various risk factors predicting or influencing disease susceptibility, treatment response, and progression to CKD among children with nephrotic syndrome.

**Trial registration:**

ClinicalTrials.gov Identifier NCT01605266.

## Background

Idiopathic nephrotic syndrome is a commonly diagnosed kidney disease in childhood and treatment resistant forms can result in scarring of the kidney, eventually progressing to chronic kidney disease (CKD) and/or end stage renal disease (ESRD) [1]. Nephrotic syndrome occurs when changes in the permselectivity barrier of the glomerular capillary wall can no longer restrict the loss of protein to a minimal level, thus resulting in massive protein loss through the urine [2]. Nephrotic syndrome can result in lethal infections, thrombosis, and pulmonary edema as a result of the significant protein loss [2]. The estimated incidence of nephrotic syndrome is between 2-7 per 100,000 children worldwide, with higher rates reported among those with African and South Asian ancestry [2-6]. The specific causes of nephrotic syndrome are disputed, but are considered to be immune-mediated based on the evidence that steroids treat the underlying disease, and on observed associations of nephrotic syndrome with atopy. Prior to the initiation of steroid treatment in the 1960s, the risk of morbidity and mortality was extremely high [7]. Most common clinical protocols provide at least 12-16 weeks of steroid therapy at diagnosis, followed by second line agents if the child is deemed steroid resistant, steroid dependant, or a frequent relapser. Current clinical convention is that the initial response to steroids will determine the long-term risk of disease progression; however, approximately 20% of children with nephrotic syndrome will not respond to steroids among those with European ancestry, and the rates of steroid treatment resistance are reported to be significantly higher among those with African (≈16-27%) and Asian (≈27-54%) ancestry (Table 1) [2-6,17,18].

**Table 1:** Observational Studies in Children with Nephrotic Syndrome

**Author**	**Year**	**N**	**Ethnicity**	**Mean Age at Onset ± SD, yrs (Range)**	**Male (%)**	**Mean Follow-Up Time ± SD, yrs (Range)**	**Steroid Resistant (%)**
**Retrospective Studies**							
Ingulli [5]	1991	177	Black & Hispanic	7.3 ± 4.6	No data	8.25 ± 4.3	15.3%
				(1.0-16.75)		(1-15)	
		65	Caucasian	7.8 ± 4.8		8.8 ± 4.1	6.2%
				(2-14.8)		(2-14.8)	
Bircan [8]	2002	138	Turkish	4.9 ± 3.56	61.2%	3.4 ± 2.31	13.2%
				(1-15)		(1-6)	
Ozkaya [9]	2004	392	Turkish	4.6 ± 3.4	59.2%	2	23%^b^
				(0.9-16)^a^			
Kim [10]	2005	103	Caucasian	4.3 ± 3.5	51%	No data	3.6%
		96	African-American	8.2 ± 5.2		No data	11%
Bhimma [3]	2006	816	Black & Indian	4.8	60.4%	2.5	27.3%
				(1.2-16)		(0.1-16.5)	
Chang [11]	2009	99	Chinese	8.35 ± 4.61	73.7%	5.06 ± 4.35	N/A^e^
				(2-18)			
Mubarak [6]	2009	538	Pakistani	9.79 ± 4.59	64.4%	No data	31.1%
				(0.8-18)^a^			
Otukesh [12]	2009	73	Iranian	5.9	52.0%	6.0 ± 4.2	100%
						(0.5-16)	
Copelovitch [4]	2010	112	Cambodian	8.95	63.4%	15.1	6.25%
				(0.6-15.75)		(3.7-2.73)	
Banaszak [13]	2012	76	Caucasian (Poland)^c^	2.7 (median)	54.5%	No data	15.8%
		102	Caucasian (Poland)^d^	3.3 (median)	68%	No data	31.3%
**Prospective Studies**							
Kumar [14]	2003	290	Northern and Eastern Indian	7.9 ± 5.1	73.4%	No data	38%^b^
Wong [15]*(Registry)*	2007	49	New Zealand European, Maori, Pacific Islander, Asian, Other	6.1 ± 3.8	71.4%	1	19.6%
Bakkali [16]*(Registry)*	2011	231	No data (Netherlands)	5.08	67.1%	4	N/A^e^

^a^ indicates age of study group, where age at onset not specified.

^b^ estimated based on participants diagnosed with FSGS.

^c^ indicates patients treated with NS between 1986-1995.

^d^ indicates patients treated with NS between 1996-2005.

^e^ no steroid resistance reported.

There have been very few longitudinal cohort studies of children with nephrotic syndrome (Table 1). Most cohort studies are retrospective chart reviews, limited by access to clinical data that have been recorded and are available. The few prospective studies on childhood nephrotic syndrome are largely registries with short follow-up time and limited clinical information. Existing cohort studies are limited to ethnically homogenous populations, which preclude the ability to test if there are true differences in treatment response among ethnicities, or if this observed difference is a result of bias from highly selected populations or differences among pediatric nephrology practices. However, this could be tested in a cohort study involving a diverse group of children treated under the same clinical protocol.

There is also an emerging picture of the role of genetics in nephrotic syndrome. In 2008, the gene, MYH9, was found to explain both the higher rates of focal segmental glomerulosclerosis (FSGS), a steroid-resistant form of nephrotic syndrome, and the higher rates of ESRD among African Americans compared to European Americans [19,20]. FSGS and ESRD were also linked to an adjacent gene on the chromosome 22 locus, APOL1, in 2010 [21]. The odds of having advanced kidney disease are 2-7 times greater for those carrying risk alleles of either MYH9 or APOL1, as compared to controls [19,20]. APOL1 has also been linked to HIV-associated nephropathy [22]. The chromosome 22 locus is also associated with both kidney disease susceptibility and kidney disease progression among Europeans; however, the allele frequency is low and thus cannot be used for clinical screening of progression [23]. Moreover, the APOL1 allele frequency varies depending on ancestry, but studies have yet to determine if those of Asian or South Asian heritage are at higher risk than those of other ethnic backgrounds [24]. Genetic factors may play a significant role in the development of disease among children, as there is limited time for exposure to non-genetic factors that influence disease risk as in adults.

It is likely that both genetic and environmental factors are involved in the onset and course of nephrotic syndrome. The onset of nephrotic syndrome has been linked to environmental influences such as mercury exposure (in adults), history of atopy, and immune response [25-28]. Renal effects have been found in children as a result of low-level exposures to cadmium, lead, mercury, and arsenic, but the role of these exposures in nephrotic syndrome has never been explicitly identified [29]. There is a small body of literature describing the associations between childhood nephrotic syndrome and socio-demographic factors, but they are mostly cross-sectional studies or prospective studies with limited follow-up, thus restricting our understanding of the determinants of health for children with nephrotic syndrome and their families due to limitations in study design and follow-up [30-39]. As a result, there is a significant gap in the literature on the role of environmental and socio-demographic modifiers of nephrotic syndrome in children in the long-term. Socio-demographic factors such as economic status, child quality of life and parental well-being, environmental factors such as exposures to lead or heavy metals, and serological modifiers, clinical factors such as hypertension or body mass index, or genetic factors may account for the variability in incidence and progression rates among various ethnic groups. A prospective cohort that addresses these variables would allow researchers to address temporal association to detect gene or gene-environment interactions that cannot be identified in case–control or cross-sectional study designs [40]. For example, if genetic screening in children could identify those that will have worse outcomes and an increased likelihood of progression, current clinical strategies will be challenged and alternative treatments to delay progression may be considered, such as the use of antihypertensive medications [41].

Insight into Nephrotic Syndrome: Investigating Genes, Health, and Therapeutics (INSIGHT) is a longitudinal study established to test for factors influencing disease susceptibility, treatment response, and progression to CKD and ESRD among children with nephrotic syndrome. Understanding the interaction between socio-demographic, environmental, clinical, and genetic factors over time is critical to developing new strategies for treating children with nephrotic syndrome. This paper describes the study design of INSIGHT, and discusses the potential implications of its research.

## Methods and study design

### Study centre

INSIGHT is a disease-based, observational longitudinal cohort study primarily based at The Hospital for Sick Children (SickKids) in Toronto, Canada. Since 1993, there has been a nurse-managed, out-patient program to teach patient and families self-monitoring and treatment of relapses from nephrotic syndrome. This program uses a validated electronic data system to track basic clinical information and relapses throughout the clinical follow-up period of each child. Staff physicians oversee the program and direct the decision-making surrounding medication dosage and patient care plans. A standard treatment protocol was implemented and developed nearly 20 years ago based on consensus of staff physicians and revised in 2000 for the treatment of relapses. The protocol involves an initial 16 weeks of steroid therapy with prednisone, comprised of a maximum of 60 mg/m^2^ per day for 6 weeks, then tapering the dose to 40 mg/m^2^ every other day for 6 weeks, 30 mg/m^2^ every other day for 8 days, 20 mg/m^2^ every other day for 8 days and finally 10 mg/m^2^ every other day for 12 days before stopping steroid treatment. A standard protocol is also used for the treatment of relapses. This is followed by second-line agents if the child is deemed steroid resistant, steroid dependant, or a frequent relapser, and is decided on a case-by-case basis.

### Aims

**Figure 1:**
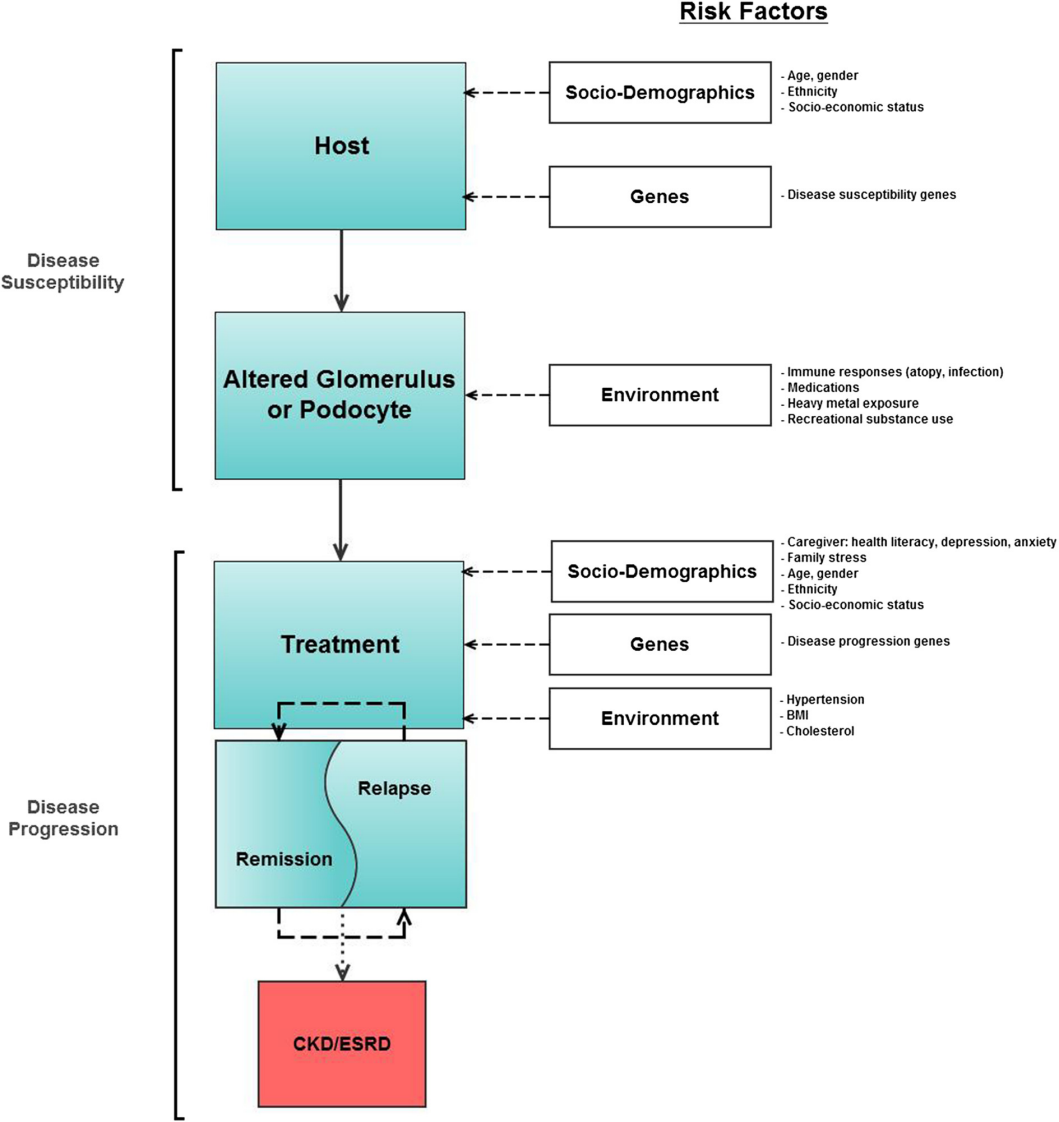
Conceptual model of nephrotic syndrome disease pathway and modifiers.

Aims of INSIGHT are to determine 1) genetic and environmental factors that influence susceptibility to nephrotic syndrome among a multi-ethnic group of children; 2) rates of steroid treatment resistance and steroid treatment dependence, and the influence of ethnicity as a treatment modifier in children with nephrotic syndrome; 3) clinical and genetic factors that influence progression of kidney disease to CKD and/or ESRD among children with nephrotic syndrome; and 4) the interaction between nephrotic syndrome and socio-demographic, environmental, and clinical modifiers (Figure 1).

### Study participants

The study population consists of incident and prevalent cases of nephrotic syndrome diagnosed or treated at SickKids after 1993. Participants concurrently followed in clinic will be seen prospectively by the study for up to five years, or until they are discharged from clinical care. Non-concurrent participants are seen once for the study. The inclusion criteria for participants are: 1) a diagnosis of presumed idiopathic nephrotic syndrome after the age of 1 and before age 18; and 2) ability to provide informed consent or have a parent or guardian provide informed consent on their behalf; and 3) the parent and child agreeing to complete questionnaires and to provide biological samples. Exclusion criteria are: 1) disease with multiple organ involvement; or 2) conditions such as systemic lupus erythematosus or vasculitis; or 3) patients with biopsy-proven membranous glomerulonephritis (MGN) or membranoproliferative glomerulonephritis (MPGN). Toronto’s ethnically and socially diverse population will allow for the recruitment of a diverse, non-homogenous cohort of children.

### Data and biological specimen collection

Baseline and follow-up questionnaires for concurrent participants are completed by the child (if able, or by the parent by proxy) and by a parent or guardian. Baseline questionnaires for the child and parent or guardian are completed upon enrollment. Follow-up questionnaires for the parent or guardian and the child are completed annually for five years, or until the child has been discharged from clinic. INSIGHT questionnaires make use of standardized questionnaires that have been tested for reliability and validity wherever possible, including the Pediatric Quality of Life Inventory (PEDSQL™-V4), the McMaster Family Assessment Device (FAD), the short version of the Patient Health Questionnaire for Depression and Anxiety (PHQ-4), and the Brief Test of Functional Health Literacy in Adults (S-TOFHLA) [42-46]. Non-concurrent participants complete a basic core questionnaire upon enrollment with no follow-up. Ethnicity is self-reported for the child with nephrotic syndrome, the biological parents and the biological grandparents (Additional file 1: Appendix 1).

Key clinical outcome data for all participants are abstracted from each participant’s electronic medical record. Set definitions are used, according to standard clinical care. Clinical outcomes include remission, relapse, frequently relapsing nephrotic syndrome, steroid dependence, and steroid resistance [47,48]. Laboratory outcomes include urine protein to creatinine ratio, urine albumin to creatinine ratio, creatinine-based eGFR, and CKD/ESRD (Table 2).

**Table 2:** Outcome Ascertainment for INSIGHT

	**Follow up time since initial onset (months)**						
	**4**	**8**	**12**	**24**	**36**	**48**	**60**
**Clinical**							
*Remission*: Urine protein/creatinine < 0.2 or Albustik negative or trace for 3 consecutive days	x	x	x	x	x	x	x
*Relapse:* After remission, an increase in the first morning urine protein/creatinine ≥2 for 3 of 5 consecutive days	x	x	x	x	x	x	x
*Frequently relapsing NS:* 4 or more relapses within 1 year OR 2 or more relapses within 6 months			x	x	x	x	x
*Steroid dependant NS*: Relapse during steroid taper or within 14 days of steroid discontinuation	x	x	x	x	x	x	x
*Steroid resistant*: Inability to induce remission within 28 days of steroid therapy	x	x	x	x	x		
**Laboratory**							
*Urine protein/creatinine*: Abnormal if > 250 mg/mmol	x	x	x	x	x	x	x
*Urine albumin/creatinine*: Abnormal if > 30 mg/mol	x	x	x	x	x	x	x
*Creatinine based eGFR*: eGFR by modified Schwartz (ml/min/1.73 m^2^) = k x height/serum creatinine	x	x	x	x	x	x	x
*CKD/ESRD*: eGFR < 60 ml/min/1.73 m^2^ (or dialysis)				Anytime			

Concurrent participants provide blood and urine samples in addition to 3 toenail clippings to test for environmental exposures (i.e. lead or other heavy metals such as mercury or cadmium). This method has previously been proven to be good at measuring exposures and is less invasive than extracting hair samples [49]. Baseline samples are collected as close to enrollment as possible and annually afterwards to coincide with follow-up. Concurrent participants will also perform a 24-hour ambulatory blood pressure monitor in their second year of the study to assess the presence of hypertension or pre-hypertension associated with nephrotic syndrome [50]. Non-concurrent participants provide biological samples at the time of enrollment. Participants who are unable to provide a blood sample may provide a saliva sample as an alternative method of DNA collection (Appendix 1).

### Data and sample management

Data are entered into REDCap™, a secure online data management portal designed for use in research [51]. All biological specimens are maintained through Freezerworks™, a biospecimen management software, and stored locally. Biological specimens are aliquoted and frozen at -80°C. This protocol was based on guidelines developed from established biobanking best practices [52]. Genomic DNA will be extracted from the blood and saliva samples obtained from participants. Blood and urine samples will also be tested for factors that may modify treatment response, such as cytokine levels, IgE levels, or complement.

### Sample size & power considerations

Our enrollment target is at least 350 participants from SickKids and the Greater Toronto Area. We have calculated the power for a minimum sample size of ~350 participants in a longitudinal analyses of time to CKD using log-rank analyses (Table 3). We demonstrate good power across the hazard ratios ranging from 1.5-3.0 and by percent exposed as compared to those unexposed to any of the potential risk factors. These values are consistent with our preliminary data of frequencies among gender and age groups of greater than 20%. Additionally, we have assumed an alpha of 0.05 and a 10% drop out in either group over a minimum of 3 years of follow-up. We have excellent power for hazards greater than 2.0 consistent with candidate genes association with FSGS such as APOL1.

**Table 3:** Power Calculation for Survival Analyses*

	**Proposed study population**								
		**350**				**400**			
	**% exposed**	**10**%	**20**%	**30**%	**40**%	**10**%	**20**%	**30**%	**40**%
**Hazard ratio**	1.5	0.31	0.59	0.74	0.8	0.37	0.66	0.8	0.85
	2	0.79	0.98	0.99	0.99	0.86	0.99	0.99	0.99
	2.5	0.98	0.99	0.99	1	0.99	0.99	1	1

*assuming an alpha of 0.05, follow-up over 3 years, and a 10% drop out in either group.

### Ethical considerations

INSIGHT has been approved by the Research Ethics Board at SickKids. Written, informed consent is obtained from each participant (the parent or guardian and the child of age to provide consent) prior to commencing any data collection for the study. All children age 13 or less provide assent to study participation in addition to parent or guardian consent. Participants are free to withdraw from the study at any time. All data collected are linked to a unique study ID number, and not to any patient identifiers. Due to the sensitive nature of biobanking DNA, only open consent is received regarding the use of DNA. INSIGHT is also a registered study under the ClinicalTrials.gov Identifier: NCT01605266.

## Results

Recruitment for INSIGHT began in 2011 at SickKids and is ongoing. As of August 13, 2012, 211 participants have been recruited into the study (Table 4).

**Table 4:** Preliminary Results from INSIGHT*

**Description**	**N**
**Child (N = 211**^**a**^**)**	
Age at diagnosis (concurrent participants)	5.34 ± 3.81 years
Age at diagnosis (non-concurrent participants)	5.22 ± 3.98 years
*Socio-Demographic Variables*	
Male	112 (60.2%)
Always speak English at home	129 (69.4%)
Participant born in Canada	163 (87.6%)
*Ethnicity*	
European	77 (41.6%)
Mixed	39 (21.1%)
South Asian (India, Sri Lanka, etc)	39 (21.1%)
Asian/Pacific Islander	15 (8.1%)
Other	15 (8.1%)
*Past Medical History*	
Gestational duration ≥ 36 weeks	149 (81.42%)
Kidney problems at birth	4 (2.2%)
History of nephrotic syndrome: mother, father, or sibling	3 (1.52%)
History of kidney disease: mother, father, or sibling	1 (0.5%)
History of kidney disease: extended family	151 (27.57%)
**Parent or Caregiver (N = 209)**	
*Socio-Demographic Variables*	
Parent use of interpreter to complete questionnaires	6 (6.4%)
Mother born in Canada	84 (45.4%)
Father born in Canada	78 (42.2%)
Families with combined income < $35,000	26 (27.66%)
Years of schooling, primary caregiver	14.5 ± 3.2 years
Age of mother at birth	31.3 ± 9.4 years

^a^ Current as of August 14, 2012, with open enrollment.

* Numbers in this table may not add up to the specified N as data are not yet available for all participants.

### Demographic characteristics

The mean participant age at onset of nephrotic syndrome is 5.28 ± 3.89 years. Males account for 60.2% of participants. Our cohort is, thus far, mostly English-speaking, with 69.4% of participants reporting that they always speak English at home.

Most study participants (n = 163, 87.6%) were born in Canada, however over half of their parents have immigrated from elsewhere. Nearly one-third of participants are classified as from a low-income family using a cut-off of $35,000, based on the Statistics Canada Low Income Measure in 2007 [53]. Primary caregivers are a well-educated group with on average, 14.5 ± 3.2 years of schooling.

### Ethnicity

Participants self-reported ethnicity for the biological grandparents, parents, and the child with nephrotic syndrome into up to four of the following categories: European/Canadian/American, South Asian, Asian/Pacific Islander, African, West Indian/Caribbean, Middle Eastern, South American, and Aboriginal. A mixed background was defined as participants reporting an ethnic ancestry in more than one category. Initial ethnic classification for 185 participants for whom self-report data are available has determined that most of the study population comes from a European ethnic background (n = 77, 41.6%), followed by participants of a mixed (n = 39, 21.1%), or South Asian (n = 39, 21.1%) background. The remaining are classified as Asian/Pacific Islander (n = 15, 8.1%) or are from other ethnic backgrounds (n = 15, 8.1%). We anticipate that as enrollment increases, the ethnic makeup of our cohort will change. This will increase generalizability to other populations not as ethnically diverse as ours in Toronto.

### Past medical history

Few participants report a history of kidney disease in the immediate family. 81.4% of participants had a full gestational term of 36 weeks or greater. Only 2.2% (n = 4) of participants report having had a kidney problem at birth. The average age of the biological mother at birth was 31.3 ± 9.4 years.

## Discussion

INSIGHT is a unique project that can: 1) address the natural history of nephrotic syndrome from the time of diagnosis, unlike other studies where participants are recruited based on biopsy-proven diagnosis or treatment response; 2) test the hypotheses of genetic risk factors for nephrotic syndrome, not yet done in a large, multi-ethnic cohort of children; and 3) increase understanding of social, clinical, and environmental factors that impact self- and family-managed chronic disease in children over the long term. The results of this study have the potential to fill these gaps in current knowledge.

Understanding how social, environmental, clinical, and genetic factors interact is important in order to truly understand the pathogenesis of disease in a diverse population of children. Nephrotic syndrome continues to be the most commonly diagnosed kidney disease in children worldwide. INSIGHT is an important and relevant project as it will challenge our current understanding of the natural history of disease in the current era of newer steroid sparing agents; in particular, the paradigm that initial response to steroid treatment is the most important indicator of disease progression [2]. Characterizing the risk of progression solely based on steroid treatment response ignores other important risk factors that are potentially modifiable and may influence overall outcome. Our current understanding of these additional risk factors is limited by previous study designs, follow-up, and use of selected populations. Furthermore, INSIGHT is a timely project due to the recent developments in genetic associations with nephrotic syndrome, and recent reports describing increased rates of disease over time [13,20,22,54,55].

Cohort studies on chronic diseases in children have allowed us to better understand natural history of disease, resulting in transformation to clinical practices. The Diabetes Chronic Complications Trial (DCCT) and its ancillary study, Epidemiology of Diabetes Interventions and Complications (EDIC), with ten years of intervention and an additional ten years of observation demonstrated that microalbuminuria could regress. This influenced how patients were counselled and treated based on the assessment of albuminuria [56,57]. Similarly, the Chronic Kidney Disease in Children (CKiD) study, currently in its sixth year and ongoing, follows children with mild to moderate chronic kidney disease for factors influencing disease progression, neurocognition, quality of life, cardiovascular health, and growth [58]. To date, CKiD has been able to develop methods for calculating glomerular filtration rate (GFR) in children more accurately, and find associations between GFR and sleep patterns, psychosocial functioning, and neurocognitive delay. These factors have not previously been recognized as significant comorbid conditions associated with early declines in kidney function [58].

Specific to nephrotic syndrome, there are a few ongoing cohort studies in progress, and it is anticipated that INSIGHT will add complementary and additional information to this body of work (see Table 5). PodoNet and the UK Registry for Rare Kidney Diseases (RaDaR) are both web-based registry studies on steroid resistant nephrotic syndrome, collect limited clinical data, and include genetic testing for known genes associated with childhood nephrotic syndrome and steroid resistance [59,60]. The Nephrotic Syndrome Study Network (NEPTUNE) is a prospective observational study where data collection begins at the time of a clinically indicated renal biopsy, after which they are followed for 30 months [61]. NEPTUNE and INSIGHT both assess quality of life and other socio-demographic and environmental factors that may influence, or be influenced by the progression of disease. PodoNet, RaDaR, and NEPTUNE all focus on those with established progressive disease sufficient to warrant a biopsy, however, at this point worse disease outcomes and factors likely influencing response may be missed. INSIGHT recruits patients from the time of diagnosis with idiopathic nephrotic syndrome, and follows the natural history of the disease course (Table 5).

**Table 5:** Summary of Prospective Studies in Glomerular Research

**Description**	**INSIGHT**	**Neptune**	**PodoNet**	**Radar**
Target N	300^a^	450	1472^b^	Not specified
# of centres	1^a^	15	85	Not specified
Length of follow-up	60 months	30 months	Not specified	Not specified
Main inclusion criteria	Presumed idiopathic nephrotic syndrome	Nephrotic syndrome-indicated renal biopsy^c^	Steroid resistant nephrotic syndrome	Steroid resistant nephrotic syndrome
***Child Data***				
Demographics	x	x		
Ethnicity & immigration	x	Not specified		
Birth history	x			
Family medical history	x			
Child allergy history	x			
Child comorbidity history (fever, viral illness, TB, jaundice, malaria)	x			
Medication adherence	x			
McMaster Family Assessment Device (FAD)	x			
Reproductive history	x			
Health behaviour and social history (i.e. substance use)	x			
Quality of life	x	x		
PROMIS Survey		x		
Beck Depression Inventory		x		
Modified Mini-Mental State Exam		x		
***Parent or Caregiver Data***				
Demographics	x			
Ethnicity & immigration	x			
Family Environment	x			
McMaster Family Assessment Device (FAD)	x			
Patient Health Questionnaire for Depression and Anxiety (PHQ-4)	x			
Pregnancy information & assessment of in-utero exposures	x			
Parent perspectives on genetic testing	x			
Shortened Test of Functional Health Literacy Assessment (S-TOFHLA)	x			
***Biorepository & Clinical Information***				
Ongoing assessment of relapses	x	Not specified		Not specified
Medications history and changes	x	x		Not specified
Standardized genetic workup			x	x
Blood collection	x	x	x	x
Urine collection	x	x	x	
Nail clipping	x	x		
Clinical progress (unspecified)	x	x	x	x

^a^ With ongoing recruitment and expansion.

^b^ Current enrollment, unspecified target.

^c^ Presumed MCNS, FSGS, MGN, MPGN.

INSIGHT is also the only study that examines the social determinants of health among children with nephrotic syndrome. The role of socio-demographic and psychosocial factors have been shown or hypothesized to influence health status among ESRD patients but there are few observational cohort studies on children to explore the role of these factors in the earlier stages of kidney disease before progression becomes a serious concern [62,63]. As nephrotic syndrome in children is largely managed at home, we are further limited by a dearth of literature on interactions between a child and caregiver. INSIGHT will help fill these gaps to help us understand socio-demographic factors that influence nephrotic syndrome, or are themselves influenced by disease progression.

There are limitations to the study which should be addressed. As with all studies collecting questionnaire data with personal and often sensitive information, some participants may be unwilling to provide information and will have limited data. This study uses validated and reliable questionnaires as much as possible in order to minimize the risk of inaccuracy and reduce the risk of measurement bias. Since not all participants will get a biopsy, we can only presume their pathological diagnosis is minimal change disease based on estimates from prior studies [17]. The strengths of INSIGHT are its large projected sample size, its lengthy projected follow-up time period, and its ethnically, socially, and clinically diverse cohort, reflecting the diverse makeup of Toronto. INSIGHT actively collects information from both the parent and the child, allowing us to generate a holistic picture of the role of the family and caregivers in the treatment of nephrotic syndrome. The open enrollment structure allows us to capture participants close to the time of clinical presentation, with the aim of being able to establish factors that truly influence the immediate progression of mild disease to more serious or chronic kidney disease.

Currently, INSIGHT is based in Toronto and efforts are underway to expand the study to several partner sites within Canada and internationally to allow for a large, ethnically diverse study population. As the study expands, we anticipate that the ethnic makeup of our cohort will become increasingly diverse, thus allowing us to better test hypotheses of ethnic associations with disease. Establishing a global, multi-ethnic cohort requires some flexibility in the core data that can reasonably collected at each site, particularly in sites in low- to middle-income countries where infrastructure to facilitate clinical research may be minimal or non-existent and follow-up of participants may be more difficult.

Understanding the interaction between socio-demographic, environmental, clinical, and genetic factors associated with disease susceptibility, steroid treatment resistance and disease progression could lead to better screening strategies at initial clinical presentation and ultimately more refined treatment strategies overall. Results from INSIGHT will lead to a better understanding of nephrotic syndrome in the current era.

## Abbreviations

ESRD: End-stage renal disease;FSGS: Focal segmental glomerulosclerosis;INSIGHT: Insight into Nephrotic Syndrome Investigating Genes, Health and Therapeutics;MGN: Membranous glomerulonephritis;MPGN: Membranoproliferative glomerulonephritis;SRNS: Steroid-resistant nephrotic syndrome;GFR: Glomerular filtration rate;NEPTUNE: Nephrotic Syndrome Study Network;DCCT: Diabetes Chronic Complications Trial;EDIC: Epidemiology of Diabetes Interventions and Complications;CKid: Chronic Kidney Disease in Children Study

## Competing interests

The authors declare that they have no competing interests.

## Authors’ contributions

RP designed the study. AZ drafted the manuscript. NH, RP, and TP revised the manuscript for important intellectual content. NH, JV, TB, and VP acquired the data. PP processes the biological specimens. RP, NH, and JV obtained funding. CB performed the statistical analysis. DH, and CL provided intellectual input and oversight. All authors read and approved the final manuscript.

## Authors’ information

NH and JV are clinical research staff at SickKids. AZ is a research program summer student. TB and VP are research students. PP is the research technologist. CB is a data analyst. DH, CL, TP and RP are all staff nephrologists at SickKids.

## Pre-publication history

The pre-publication history for this paper can be accessed here:

http://www.biomedcentral.com/1471-2369/14/25/prepub

## Supplementary Material

Additional file 1: Appendix 1 Schedule of Data and Specimen Collection by Cohort and Visit. Click here for file
