# Supplementary material for: The rationale and design of Insight into Nephrotic Syndrome: Investigating Genes, Health and Therapeutics (INSIGHT): a prospective cohort study of childhood nephrotic syndrome
Source: BMC Nephrol. 2013 Jan 26;14:25. doi: 10.1186/1471-2369-14-25 (PMC3608224; doi:10.1186/1471-2369-14-25)
Supplement: Additional file 1: Appendix 1 — Schedule of Data and Specimen Collection by Cohort and Visit. [file 1471-2369-14-25-S1.docx]

| **Appendix 1**: Schedule of Data and Specimen Collection by Cohort and Visit | | | | | | | | | | | | |
| --- | --- | --- | --- | --- | --- | --- | --- | --- | --- | --- | --- | --- |
|  | | Non-concurrent | Concurrent | | | | | | | | | |
|  |  |  | Baseline (V1) | | 12 month (V2) | | 24 Month (V3) | | 36 Month (V4) | | 48 Month (V5) | |
|  |  |  | Parent | Child | Parent | Child | Parent | Child | Parent | Child | Parent | Child |
| *Questionnaire* | | | | | | | | | | | | |
| *Informed consent* | | x | x | x |  |  |  |  |  |  |  |  |
| *Contact information* | | x | x | x |  |  |  |  |  |  |  |  |
| *Ethnicity and immigration* | | x | x | x |  |  |  |  |  |  |  |  |
| *Socio-demographic data* | | x | x | x | x |  | x |  | x |  | x |  |
| *Family Environment and McMaster Family Assessment Device (FAD)* | |  | x | x^a^ | x | x^a^ | x | x^a^ | x | x^a^ | x | x^a^ |
| *Patient Health Questionnaire for Depression and Anxiety (PHQ-4)* | |  | x |  | x |  | x |  | x |  | x |  |
| *Pregnancy Information* | |  | x |  |  |  |  |  |  |  |  |  |
| *Perspectives About Genetic Testing* | | x | x |  |  |  |  |  |  |  |  |  |
| *Health Literacy and Numeracy (S-TOFHLA)* | |  | x |  |  |  |  |  |  |  |  |  |
| *Travel History* | |  |  | x |  |  |  |  |  |  |  |  |
| *Birth History* | | x |  | x |  |  |  |  |  |  |  |  |
| *Family Medical History* | | x |  | x |  | x |  | x |  | x |  | x |
| *Allergy, Infection, and Co-Morbidity History* | |  |  | x |  |  |  |  |  |  |  |  |
| *Medications History and Adherence* | |  |  | x |  | x |  | x |  | x |  | x |
| *Reproductive History^b^* | | x |  | x |  | x |  | x |  | x |  | x |
| *Health Behaviours and Social History a* | |  |  | x |  | x |  | x |  | x |  | x |
| *Pediatric Quality of Life Inventory (PEDSQL^TM^-V4)* | |  |  | x |  | x |  | x |  | x |  | x |
| *Biological Specimen Collection* | | | | | | | | | | | | |
| *Toenails* | |  |  | x |  |  |  |  |  |  |  |  |
| *Blood* | | x |  | x |  | x |  | x |  | x |  | x |
| *Urine* | | x |  | x |  | x |  | x |  | x |  | x |
| *Saliva* | | Collected when required in order to facilitate DNA collection when blood work is not available. | | | | | | | | | | |
| *Biopsy* | | n/a | Additional visits in the year of the biopsy | | | | | | | | | |
| *Clinical Measurements* | | | | | | | | | | | | |
| *24-hour Ambulatory Blood Pressure* | |  |  |  |  | x |  |  |  |  |  |  |
| **Legend:** | ^a^ indicates on questionnaires for ages 8+ only  ^b^ indicates for females only | | | | | | | | | | | |
